# Long-Term All-Cause Mortality After Acute Pancreatitis: Median 7-Year Follow-Up

**DOI:** 10.5152/tjg.2025.25575

**Published:** 2025-11-21

**Authors:** Elmas Biberci Keskin, Bahadır Taşlıdere, Sercan Kiremitçi, İbrahim Hakkı Köker, Özlem Toluk, Hakan Şentürk

**Affiliations:** 1 Department of Internal Medicine, Health Sciences University, Hamidiye Etfal Education and Research Hospital, İstanbul, Türkiye; 2 Department of Emergency Medicine, Bezmialem Vakıf University Faculty of Medicine, İstanbul, Türkiye; 3 Department of Gastroenterology, Başkent University, Türkiye; 4 Department of Biostatistics and Medical Informatics, Bezmialem Vakıf University, İstanbul, Türkiye; 5 Department of Gastroenterology, Bezmialem Vakıf University Faculty of Medicine, İstanbul, Türkiye

**Keywords:** Acute pancreatitis, complication, mortality

## Abstract

**Background/Aims:**

Acute pancreatitis is a life-threatening disease associated with significant in-hospital mortality, particularly when complications occur. However, there is little data for long-term all-cause mortality of acute pancreatitis and its relation to etiology. Thus, in this study, both short- and very long-term all-cause mortality after acute pancreatitis were sought.

**Materials and Methods:**

Patients admitted with acute pancreatitis to the Bezmialem Vakıf University Hospital from 2012 to 2023 were retrospectively investigated. Demographic and laboratory values were obtained from hospital records. All-cause mortality was assessed using the national death notification system.

**Results:**

: A total of 689 patients were included in the final analysis. The median follow-up duration was 86 months (range: 0-154 months). The mean age of the study population was 54.3 ± 16.9 years, and 384 individuals (55.7%) were women. The overall in-hospital mortality rate was 2.8%, while 1-year all-cause mortality was 5.4%. Age, diabetes mellitus, and development of in-hospital complications were independent predictors of all-cause long-term mortality. In terms of etiology, alcoholic and hypertriglyceridemia-induced acute pancreatitis had higher in-hospital mortality rates; however, over the long term, alcoholic pancreatitis yielded the worst prognosis. The first year after discharge showed the highest mortality that is most likely associated with in-hospital complications and disease severity.

**Conclusion:**

This study sheds light on modern-era mortality rates of acute pancreatitis. Age, diabetes, and in-hospital complication development had a significant impact on long-term survival. Although underrepresented in this cohort, alcoholic pancreatitis had the highest long-term all-cause mortality and clearly represents an issue that deserves to be investigated.

**Main Points**

- Overall, the 1-year all-cause mortality rate is approximately 5% for acute pancreatitis.
- Age, diabetes, and in-hospital complications are strong predictors of mortality.
- Alcoholic pancreatitis had the highest long-term mortality rate, reaching up to 22%.
- The first year represents the most vulnerable period.

## Introduction

Acute pancreatitis is a gastrointestinal inflammatory disease that requires urgent hospitalization. According to the latest epidemiologic studies, its incidence is rising mainly due to gallstones and alcohol misuse, and is around 30 cases per 100 000 patients.^1^ Although it usually presents as a mild and self-limiting form, approximately 20% of cases develop moderate to severe pancreatitis, which is characterized by pancreatic or peripancreatic necrosis.^2^ Especially when complications occur and organ failure develops, it turns into a life-threatening disease with significant in-hospital mortality. Generally, the in-hospital mortality rate ranges from 2.7% to 7.5% depending on the characteristics of the cohort studied.^3^ Acute pancreatitis-related in-hospital death usually occurs within the first 2 weeks of admission, reflecting the rapid progression of organ failure.

Beyond in-hospital complications, permanent organ damage and accompanying comorbidities continue to affect the prognosis of these patients, leading to recurrent attacks and hospitalizations. However, previous research, including randomized controlled trials, mainly focused on the in-hospital and early discharge period. Most scoring systems available are basically designed to predict the severity and thus in-hospital complications of the disease, lacking long-term mortality prediction. Studies with a longer follow-up generally have limited sample sizes or shorter durations of follow-up.^4^ In fact, there is limited data with respect to long-term survival after acute pancreatitis. Thus, in this study, the very long-term survival of patients with acute pancreatitis and factors related to mortality were sought to be determined. Also, the current trends in outcomes with modern therapy were investigated.

## Materials and Methods

### Patients

This study retrospectively examined the demographic and laboratory profiles of patients admitted with acute pancreatitis during the period from January 2013 to December 2018. Cases lacking sufficient data were excluded. Acute pancreatitis was diagnosed in accordance with the revised Atlanta criteria, requiring 2 or more of the following: typical abdominal pain, amylase/lipase elevation greater than 3-fold the normal value, or imaging findings consistent with the disease.^5^ Ethical clearance was obtained from Bezmialem Vakıf University Ethics Committee (Number: 2025/49; Date: February 19, 2025). Written informed consent was obtained from all participants who agreed to take part in the study.

Disease severity was graded according to the Japanese Severity Score (JSS), an established tool combining clinical and radiological criteria. Details of the scoring methodology have been reported elsewhere.^6^

### In-Hospital Complications

Pseudocyst formation, abscess, walled-off necrosis, necrosis, sepsis, and pleural effusion were defined as in-hospital complications.

### Outcome

The primary endpoint of the study was all-cause mortality. In-hospital deaths were identified through hospital records. The patients were evaluated after discharge during outpatient visits. Those who did not come to routine outpatient visits were interviewed by telephone, and lastly, each patient’s final status was checked using the national death notification system.

### Statististics

Continuous data were summarized as mean ± SD or median (interquartile range, IQR) depending on distribution, and categorical data as counts with percentages. Normality was tested with the Kolmogorov–Smirnov test. Between-group comparisons were made using the t-test or Mann–Whitney *U-*test for continuous variables and Chi-square or Fisher’s exact test for categorical variables. Survival was analyzed by log-rank testing, and predictors of all-cause mortality were explored using Cox regression. Variables included in multivariable analysis were selected based on clinical importance or univariate *P* < .15. Statistical significance was set at *P* < .05 (two-tailed). All analyses were performed in R (v4.4.1) (R Foundation; Vienna, Austria).

## Results

Overall, 689 patients were included in the study. Baseline demographics, laboratory values, and in-hospital complications are presented in Table 1. The mean age of the cohort was 54.3 ± 16.9, and 384 (55.7%) patients were female. The average age of the patients who died during follow-up was significantly higher compared to those who survived (69.1 ± 15.0 vs 51.0 ± 15.4, *P* < .01). Likewise, diabetes mellitus was more common in the patients who died (n = 44 [35.2%] vs n = 110 [19.5%], *P* < .01). In terms of etiology, the most common etiology was biliary, and there was no statistical mortality difference among several etiologies. Both the rate of severe acute pancreatitis (based on both Japanese classification and Bedside Index for Severity in Acute Pancreatitis [BISAP] scoring) and the rate of in-hospital complications were significantly higher in patients who died during follow-up (62.9% vs 39.5% and 34.4% vs 16.8%, *P* < .01, respectively). The most common complication was necrosis of the pancreas, which was followed by pseudocyst formation. Similarly, intensive care unit admission was more common in this group. The cholecystectomy rate was significantly higher in patients who survived (n = 136 [24.1%] vs n = 18 [14.4%], *P* = .02). Serum calcium levels were lower in patients who died (8.9 ± 1.2 vs 9.1 ± 0.7, *P* = .02).

**Figure 1.**
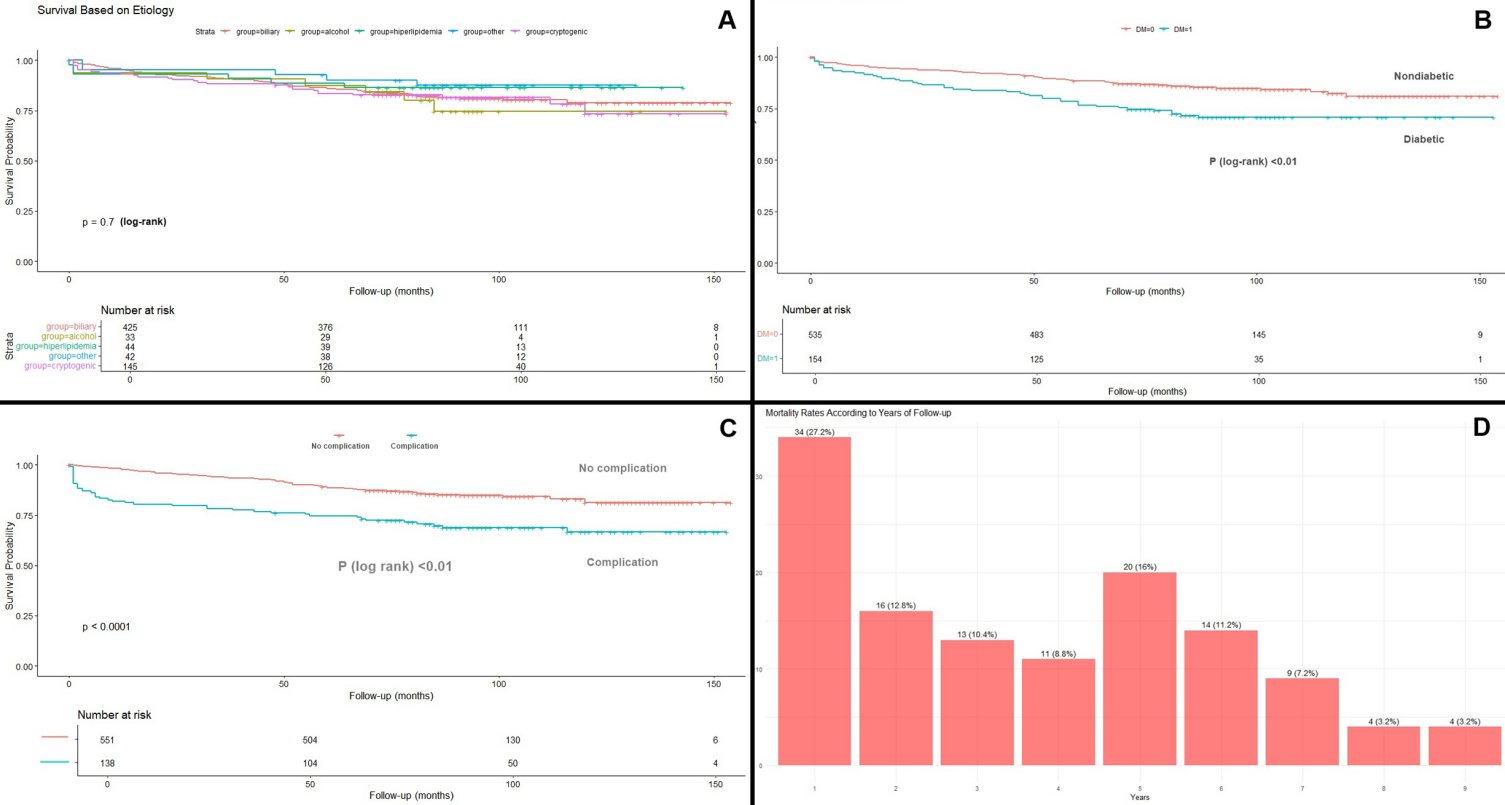
A: Kaplan–Meier analysis shows survival according to acute pancreatitis etiology. B: Survival analysis according to the presence of diabetes mellitus. C: Survival analysis according to the development of in-hospital complications. D: Mortality rates across several years of follow-up.

Mortality rates according to etiology are given in Table 2. Overall, in-hospital, 30-day, 1-year, and long-term mortality rates were 2.5%, 3.2%, 5.4%, and 18.1%, respectively. Over the long term, the highest mortality was seen in alcoholic pancreatitis (21.7%); however, this did not reach statistical significance. Figure 1A demonstrates Kaplan–Meier survival curves based on etiology (*P* = .71). Median follow-up was 86 months (0-154 months).

**Figure 2.**
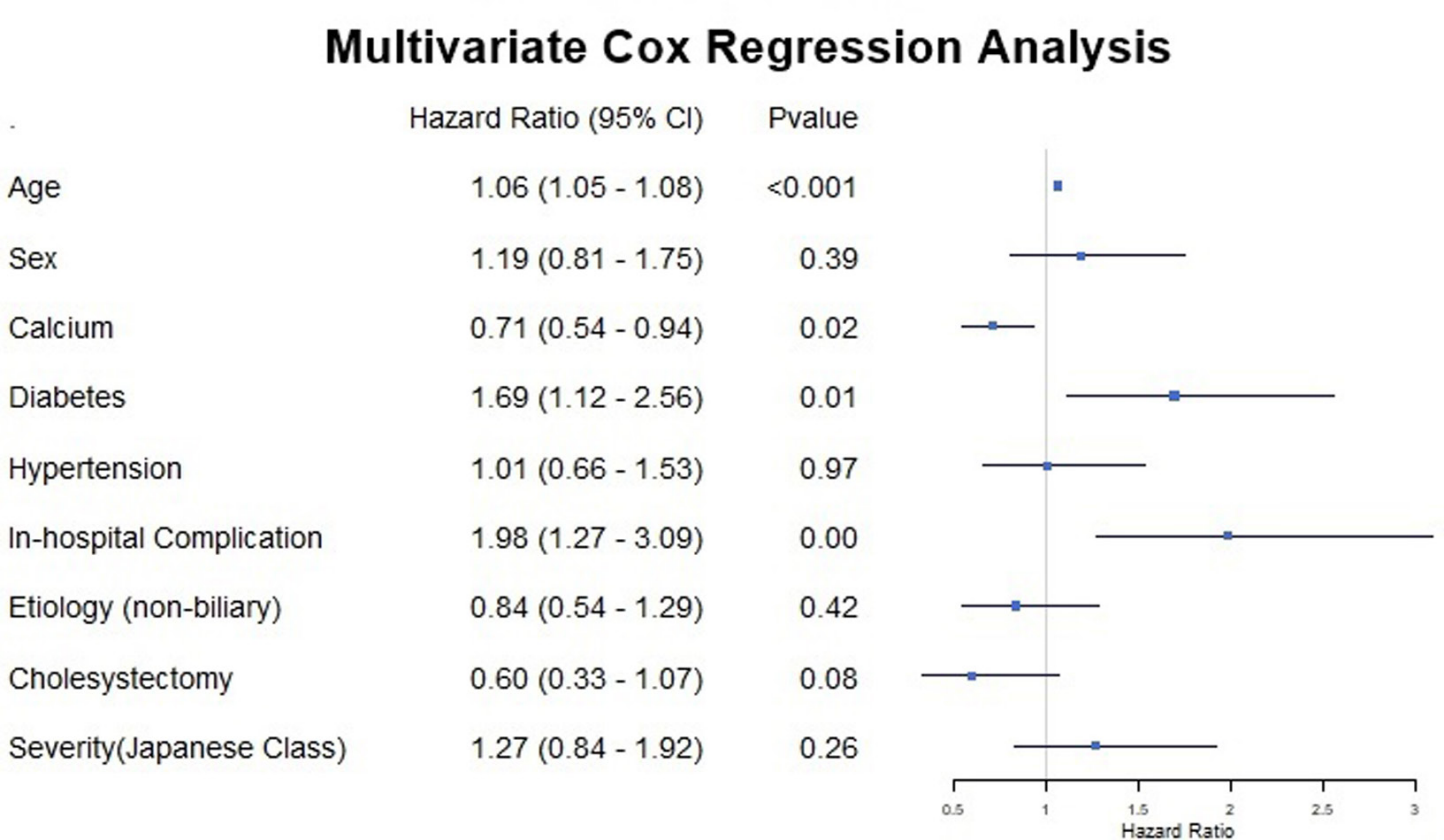
Forest plot depicting multivariate Cox regression analysis.

**Figure 3.**
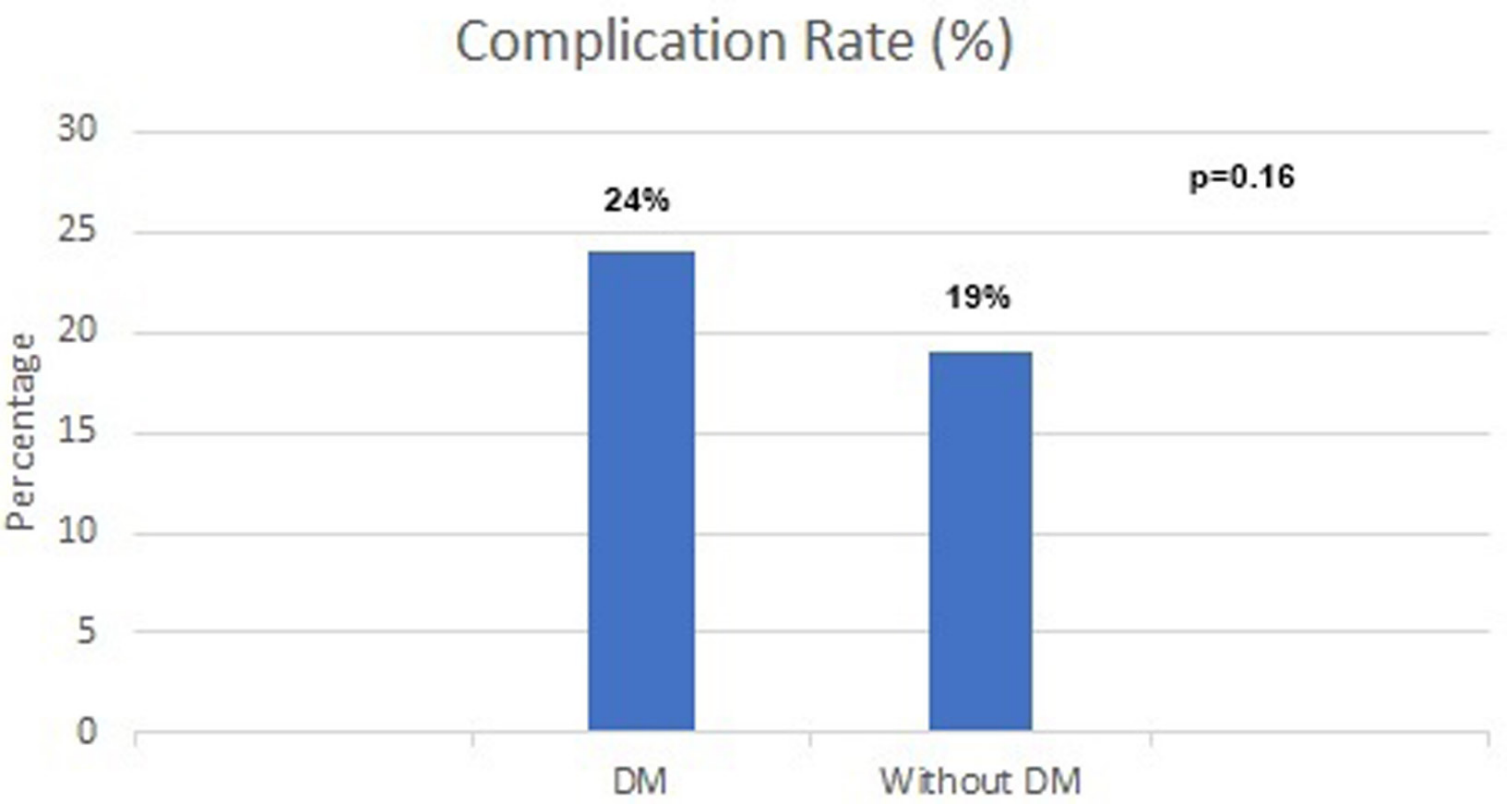
Complication rates of diabetic and nondiabetic patients.

In Table 3, the results of the univariate Cox regression analysis are presented. Age, diabetes mellitus, hypertension, cholecystectomy, in-hospital complications, serum calcium, and Japanese severity score all revealed statistical significance. However, when multivariable analysis was performed, only age (hazard ratio [HR] = 1.06, [95% CI = 1.05-1.08], *P* < .01), diabetes mellitus (HR = 1.69, [95% CI = 1.12-2.56], *P* = .01) and in-hospital complications (HR = 1.98, [95% CI = 1.27-3.09], *P* < .01) were found to be independent predictors of mortality (Figure 2). Figures 1B and 1C depict Kaplan–Meier survival analysis of patients with diabetes and in-hospital complications and show the trend during the follow-up. Figure 1D demonstrates yearly mortality rates during the follow-up. The highest mortality rate was seen during the first year (n = 34 [27.2%]). Diabetic patients had higher in-hospital complication rates, but this did not reach statistical significance (Figure 3).

## Discussion

In this study, the in-hospital mortality rate was 2.8%, 30-day all-cause mortality was 3.2% and 1-year all-cause mortality was 5.4%. Age, diabetes mellitus, and in-hospital complications were independent predictors of mortality. The current findings seem to be parallel with the previous studies. For instance, in a large study by Davidsen et al,^7^ the in-hospital mortality rate was 3.3%, and the early (90-day) post-discharge mortality rate was 5% for patients with acute pancreatitis.

Age is a risk factor for mortality in general. In terms of pancreatitis, several studies identified age as a risk factor. For instance, Husu et al^8^ investigated patients who were admitted to the intensive care unit with acute pancreatitis, with a follow-up reaching up to 17 years. In their study, patients over 60 years of age had a higher mortality rate.^8^ Similarly, Czapari et al^9^ studied over 2600 patients with pancreatitis, in which age was one of the predictors of mortality after discharge. Knudsen JS et al investigated the trends in incidence and mortality of acute pancreatitis in a 31-year follow-up study, and they once again demonstrated that age was one of the predictors of mortality.^10^ Apart from that, Lee et al conducted a similar survival study in patients with acute pancreatitis and found that the Charlson comorbidity index, higher severity index score, and length of hospital stay were also predictors of mortality.^22^

The relationship between acute pancreatitis and diabetes mellitus is complex. First of all, experimental studies showed that pre-existing diabetes worsens the severity of acute pancreatitis. Zechner et al^12^ showed that diabetes increased plasma interleukin 6 concentration and decreased lymphocyte levels during acute pancreatitis. Thus, diabetes may exacerbate pancreatitis-induced systemic inflammation. There are some other studies that showed increased pancreatic fibrosis and limited regeneration.^13^ Second, the degree of glycemic control has a significant interplay with the course of acute pancreatitis. The presence of hyperglycemia has been shown to be closely associated with higher mortality and morbidity in acute pancreatitis.^14^ In addition, diabetic patients have an increased propensity for hypertriglyceridemia and gallstones, both of which may lead to acute pancreatitis. Third, most patients with diabetes have comorbid conditions such as heart failure, chronic renal failure, and obesity, all of which also have a significant impact on long-term survival. Nevertheless, the presence of diabetes, regardless of the mechanism behind it, has a negative impact on survival.

One other important factor that was related to increased mortality was the development of in-hospital complications, which is merely a reflection of the severity of the disease course. In this cohort, 20% had in-hospital complications, of which necrosis was the most common. Approximately 10%-20% of patients with acute pancreatitis develop pancreatic or peri-pancreatic necrosis.^15^^,^^16^ While most cases of necrosis remain sterile, infection may accompany in approximately 30% of patients.^17^

There are many risk-scoring systems available to predict the prognosis of acute pancreatitis. In this study, both JSS and BISAP were used to distinguish severe pancreatitis, which in turn both predict poor prognostic factors such as complications, intensive care unit admission, and mortality. Although both scoring systems have been widely adopted, JSS has the advantage of incorporating both clinical and radiological findings and has been validated before.^18^ For instance, in order to evaluate the ability of the JSS to predict the severity of acute pancreatitis, in-hospital mortality rates were analyzed in a study by Hamada et al^19^ involving a total of 17 901 patients. The in-hospital mortality rate was 2.6%, and the mortality rates of non-severe and severe acute pancreatitis were 1.1% and 7.0%, respectively. In this study, while approximately 40% of the cohort was classified as severe, despite that, the in-hospital mortality rate was 2.8%, which may be considered relatively low. There may be several explanations for these findings. Compared to previous older data, changes in the recommendations of the relevant guidelines might have led to better outcomes. Likewise, the center is an experienced tertiary care facility to which many acute pancreatitis patients are referred. Thus, it may differ from other studies that included heterogeneous healthcare facilities.

In terms of etiology, although the long-term mortality rate was numerically highest in alcohol-related acute pancreatitis, it did not reach statistical significance due to limited sample size. First off, patients with alcoholic pancreatitis were underrepresented in the current cohort compared to Western countries. Thus, it may be hard to draw solid conclusions. Nevertheless, this finding is consistent with the current literature. For instance, in an interesting study of 1644 patients with acute pancreatitis in which alcohol consisted of 71% of the cases, the mortality rate was 24% during a 9.5-year follow-up.^20^ In general, the long-term mortality rate seems to be 4 times higher than that of the general population.^20^ The reason for higher mortality may be a lack of patient compliance with treatment and other lifestyle recommendations, or associated with alcohol-related comorbidities. However, the incidence of mortality changes during the initial hospital admission period, with hypertriglyceridemia-induced acute pancreatitis showing the highest in-hospital mortality, reaching up to ~7%. This finding is most likely associated with the severity rather than the etiology, as approximately 55% of these patients were classified as severe based on the Japanese classification.

Overall, the highest mortality was seen within the first year, almost doubling the number of in-hospital deaths. This trend most likely represents early complications related to pancreatitis. Similarly, in a study by Czapari et al,^9^ the 12-month post-discharge mortality rate was 5.5% and 3% within the first 90 days. Thus, 55% of the total 1-year mortality occurred in the first 90-day period. In a population-based study by Davidsen et al,^7^ the hazard ratio for 90-day mortality after discharge was 7.62, showing a net increased mortality risk. Thus, the early post-discharge period seems to be the most dangerous time interval. Cardiovascular and gastrointestinal complications were held responsible for early mortality in previous studies. One important study showed that patients discharged after acute pancreatitis had an almost eightfold increased risk of death in the 90-day post-discharge period compared with a matched cohort from the general population.^21^

### Limitations

This was a retrospective single-center study, and because of its observational design, hidden confounding factors may remain. Pancreatitis-related mortality was not specifically investigated, as there might be so many competing risks. Thus, in order to overcome that, all-cause mortality was assessed. Although diabetes was an independent risk factor for mortality, the impact of adequate diabetes control on patient survival was not particularly assessed. These results reflect the demographics of an experienced university hospital practice, which may not be representative of the whole region. The inclusion period was spread over a decade during which changes in treatment guidelines and patient demographics may have affected the results. Finally, the lack of a control group may be considered a limitation, even though it may be hard to establish a truly healthy population that matches baseline characteristics.

In conclusion, in this study, the in-hospital mortality rate was 2.8%, 30-day all-cause mortality was 3.2% and 1-year all-cause mortality was 5.4%. Age, diabetes mellitus, and in-hospital complications were independent predictors of mortality. Alcohol-induced acute pancreatitis had the highest long-term mortality rate. The first year after discharge is the time with the highest mortality, most likely associated with in-hospital complications and disease severity.

## Figures and Tables

**Table 1.** Baseline Clinical Characteristics and Laboratory Values of the Study Patients

		**All Patients** **N = 689, n (%)**	**Survived** **N = 564 (81.7%), n (%)**	**Died** N = **125 (18.1%), n (%)**	***P***
Age (years)		54.3 (16.9)	51.0 (15.4)	69.1 (15.0)	**<.01**
Sex (female)		384 (55.7)	320 (56.7)	64 (51.2)	.30
Diabetes mellitus		154 (22.4)	110 (19.5)	44 (35.2)	**<.01**
Hypertension		127 (21.7)	92 (19.5)	35 (31.0)	**.01**
BMI (kg/m^2^)		28.7 (5.5)	28.9 (5.5)	27.7 (5.6)	.06
Cholecystectomy		154 (22.4)	136(24.1)	18 (14.4)	**.02**
Etiology				
Biliary	425 (61.7)	346 (61.3)	79 (63.2)	.72
	Idiopathic	145 (21.0)	117 (20.7)		28 (22.4)
	Hyperlipidemia	45 (6.5)	39 (6.9)		6 (4.8)
	Alcohol	33 (4.8)	26 (4.6)		7 (5.6)
	Other	41 (6.0)	36 (6.4)		5 (4.0)
Multiple attacks		187 (21.1)	158 (28.0)	29 (23.2)	.32
AST (IU/L)		87 (23-254)	96 (24-266)	69 (23-192)	.12
ALT (IU/L)		90 (23-267)	104 (25-294)	55 (18-268)	**<.01**
ALP (IU/L)		115 (78-187)	115 (78-187)	108 (78-188)	.82
GGT (IU/L)		153 (47-319)	162 (45-336)	117 (50-265)	.28
Direct bilirubin (mg/dL)		0.5 (0.2-1.4)	0. 5(0.2-1.5)	0.5 (0.2-1.2)	.81
Total bilirubin (mg/dL)		1.1 (0.6-2.2)	1.1 (0.6-2.3)	1.1 (0.6-2.0)	.99
Amylase (mg/dL)		838 (323-1840)	838 (327-1970)	821 (265-1604)	.58
Lipase (mg/dL)		1694 (573-4485)	1736 (582-4485)	1296 (504-4623)	.48
Calcium (mg/dL)		9.1 (0.8)	9.1 (0.7)	8.9 (1.2)	**.02**
Triglyceride (mg/dL)		96 (65-145)	95 (65-150)	99 (63-138)	.79
INR		1.1 (0.6)	1.1 (0.7)	1.2 (0.5)	**.02**
WBC (×10^3^)		12.4 (5.4)	12.2 (5.2)	13.3 (5.9)	.07
Hematocrit (%)		39.8 (6.1)	40.0 (5.9)	39.0 (6.9)	.14
CRP (mg/dL)		1.8 (0.6-7.2)	1.7 (0.6-6.6)	2.6 (0.7-9.8)	.06
Glucose (mg/dL)		140 (62)	136 (59)	162 (71)	**<.01**
Creatinine (mg/dL)		0.9 (0.5)	0.8 (0.5)	1.0 (0.6)	**<.01**
JSS		2.7 (1.4)	2.5 (1.3)	3.5 (1.6)	**<.01**
Severe acute pancreatitis (based on JSS)		296 (43.8)	218 (39.5)	78 (62.9)	**<.01**
BISAP score		1.2 (0.9)	1.0 (0.9)	1.9 (0.9)	**<.01**
Severe AP (based on BISAP)		93 (13.5)	50 (8.9)	43 (34.4)	**<.01**
Intensive care admission		51 (7.4)	29 (5.1)	22 (17.6)	**<.01 **
In-hospital complications, n (%)		138 (20.0)	95 (16.8)	43 (34.4)	**<.01**
Pseudocyst		38 (5.5)	30(5.3)	8 (6.4)	
WON		9 (1.3)	6 (1.1)	3 (2.4)	
Abscess		7 (1.0)	5 (0.9)	2 (1.6)	
Necrosis		61 (8.9)	44 (7.8)	17 (13.6)	
Sepsis		9 (1.3)	1 (0.2)	8 (6.4)	
Other		14 (2.0)	9 (1.6)	5 (4.0)	
ERCP		283 (41.1)	226 (40.1)	57 (45.6)	.30
In-hospital infection		69 (10.0)	54 (9.6)	15 (12.0)	.51

ALP, alkaline phosphatase; ALT, alanine aminotransferase; AST, aspartate aminotransferase; AP Acute Pancreatits, BISAP, Bedside Index for Severity in Acute Pancreatitis; BMI, body mass index; CRP, C-reactive protein; ERCP, endoscopic retrograde cholangiopancreatography; GGT, gamma-glutamil transferaz; INR, international normalized ratio; JSS, Japanese Severity Score; WBC, white blood cell; WON, walled-off necrosis.

Bold numbers represent statistical significance.

**Table 2.** Mortality Rates According to Etiology

	**In-Hospital Mortality** **n = 19 (2.8%)**	**30-Day All-Cause Mortality** **n = 22 (3.2%)**	**1-Year All-Cause Mortality** **n = 37 (5.4%)**	**Long-Term All-Cause Mortality** **n = 125 (18.1%)**
Biliary (n = 425), n (%)	9 (2.1)	12 (2.8)	20 (4.7)	79 (18.6)
Idiopathic (n = 145), n (%)	5 (3.4)	5 (3.4)	10 (6.9)	28 (19.3)
Hyperlipidemia (n = 45), n (%)	3 (6.8)	3 (6.8)	3 (6.8)	6 (13.3)
Alcohol (n = 33), n (%)	2 (6.1)	2 (6.1)	2 (6.1)	7 (21.7)
Other	0	0	2 (4.8)	5 (11.9)
*P*	0.18	0.36	0.86	0.71

**Table 3.** Cox Proportional Hazard Regression Analysis

**Univariate Analysis**			
	**HR**	**95% CI**	***P***
Age	1.06	1.05-1.08	**<.01**
Sex (male)	1.22	0.86-1.73	.25
Recurrence	0.74	0.49-1.12	.16
Etiology (biliary as reference)	0.94	0.65-1.35	.76
DM	2.02	1.39-2.91	**<.01**
HT	1.78	1.19-2.65	**<.01**
Cholecystectomy	0.53	0.32-0.88	**.01**
Complication	2.33	1.61-3.38	**<.01**
Serum calcium	0.66	0.53-0.82	**<.01**
Triglyceride	1.00	1.00-1.00	.95
CRP (48 hrs)	1.00	0.99-1.01	.21
Amylase	1.00	1.00-1.00	.55
ERCP	1.21	0.85-1.73	.27
Infection	1.26	0.74-2.17	.38
Japanese Severity Score	2.38	1.65-3.43	**<.01**
HCT	0.97	0.49-1.01	.10

CRP, C-reactive protein; DM, diabetes mellitus; ERCP, endoscopic retrograde cholangiopancreatography; Hct, haematocrit; HT, hypertension.

Bold numbers represent statistical significance.

## Data Availability

The data that support the findings of this study are available on request from the corresponding author.
